# CHK1 as a Metabolic and Immunological Regulator: Implications for Cancer Therapy

**DOI:** 10.32604/or.2026.076509

**Published:** 2026-05-21

**Authors:** Maria Franza, Aurora Melfi, Filippo Acconcia, Alessandra di Masi

**Affiliations:** Department of Science, Roma Tre University, Rome, Italy

**Keywords:** Checkpoint kinase 1 (CHK1), metabolism, glycolysis, immunomodulation, cancer therapy, checkpoint inhibition, synthetic lethality, immune evasion

## Abstract

Checkpoint kinase 1 (CHK1), a key regulator of cell cycle checkpoints, plays a central role in the DNA damage response network, serving as a critical mediator that links DNA damage detection to DNA repair mechanisms. In recent years, several other cellular functions of CHK1 have gradually been discovered. As well as monitoring genomic integrity, CHK1 coordinates the timing of DNA replication with the availability of metabolic resources. This prevents unscheduled DNA synthesis from exceeding the cell’s metabolic capacity and causing DNA damage. CHK1 activity also contributes to tumour immune surveillance and the modulation of immune cell infiltration and immune escape mechanisms within the tumour microenvironment. Furthermore, CHK1 is involved in the regulation of differentiation and epigenetics. This perspective on CHK1 provides a strategy foundation for next-generation combinatorial therapies. Indeed, the data presented herein underscores the potential of novel therapeutic strategies that target diverse aspects of tumour biology in a simultaneous manner. Despite the current lack of clinically approved CHK1 inhibitors, the pleiotropic roles of this kinase make it an attractive and promising target for new cancer therapies. The present review aims to analyze the structural and functional aspects of CHK1, with a particular focus on its “non-canonical” functions.

## 1 Introduction

Checkpoint kinase 1 (CHK1) is a pivotal regulator of cell cycle checkpoints and represents a central component of the DNA damage response (DDR) network. CHK1 acts as a critical intermediary integrating signals from DNA damage sensors and orchestrating downstream repair pathways to maintain genomic integrity [1]. CHK1 precisely schedules DNA replication events to match the cell’s metabolic capacity, thereby preventing detrimental replication stress (RS) and avoiding excessive DNA damage caused by unscheduled synthesis [2]. CHK1 kinase activity controls this function, enabling CHK1 to phosphorylate various downstream targets to inhibit origin firing during replication and stabilise replication forks. This maintains genome integrity [3,4].

CHK1 plays an essential role in cell cycle control that is independent of its canonical function in DDR and genome integrity maintenance [5]. CHK1 primarily acts as a safety mechanism blocking proliferating cells during all the cell cycle phases (i.e., G1, S, G2, and mitosis), thus regulating the cellular decision to either progress or stall at specific checkpoints. This ensures that damaged or incompletely replicated DNA is not passed on to daughter cells. The role of CHK1 in cell cycle progression is physiological, independent of exogenous stress or DNA damage [5,6].

Recent findings have revealed a plethora of non-canonical cellular functions for CHK1. This kinase has been shown to modulate chromatin structure through histone modifications, which represent an intrinsic control mechanism that contributes to the coordination of cell-cycle progression with the activation of transcriptional programs. This, in turn, supports tissue homeostasis during physiological development [7,8,9,10,11]. CHK1 also plays an important role in the modulation of the tumour microenvironment (TME) [12,13].

**Figure 1:**
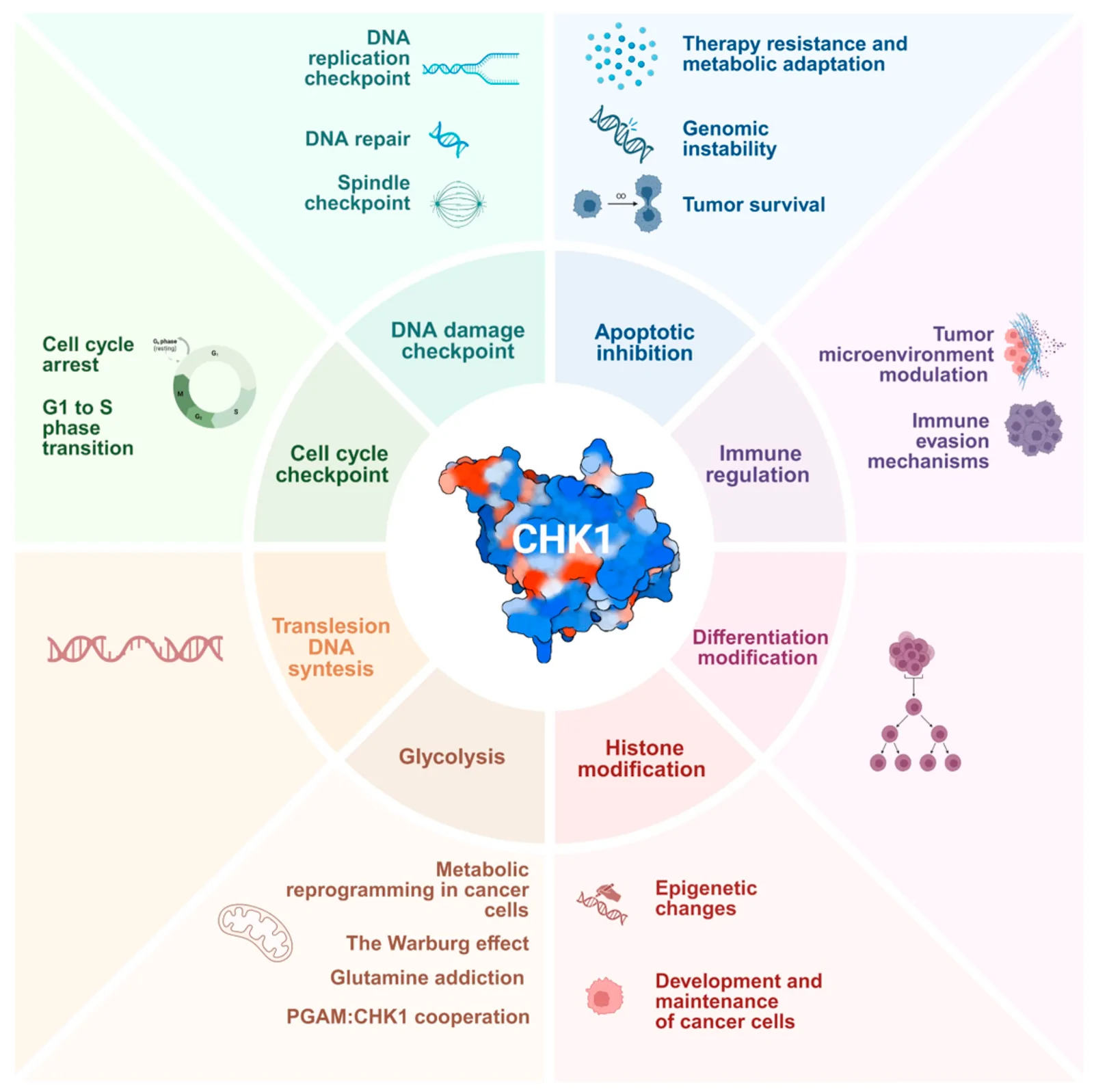
Canonical and non-canonical functions of the CHK1 kinase. Schematic illustration of the cellular processes regulated by CHK1 kinase, emphasising its multifunctional role in responding to DNA damage, maintaining genomic integrity, and controlling cellular metabolism and epigenetic modifications. CHK1 is also involved in inhibiting apoptosis and modulating cellular differentiation and immune processes. The side panels provide a more detailed description of the main downstream mechanisms modulated by CHK1 activity. Created in https://BioRender.com.

The aim of this review is to summarise the literature pertaining to CHK1-associated cross-talk between DDR, chromatin modifications, the immune system, and cellular metabolism. This allowed us to propose a paradigm shift in CHK1 functions by redefining this kinase as a multifaceted regulator, moving from its traditional role in cell cycle regulation to new functions in metabolic resilience and immune escape (Fig. 1). A synthesis of the extant evidence on the use of anti-cancer therapies combining CHK1 inhibitors (iCHK1) with immunological or metabolic agents is also presented. This new perspective on CHK1 provides a strategy foundation for next-generation combinatorial therapies. Indeed, data reported highlights the potential of new therapeutic strategies that target different aspects of tumour biology simultaneously. Notwithstanding the paucity of clinically approved iCHK1 to date, the pleiotropic roles of this kinase in metabolic and immune regulation render it an attractive and promising target for new cancer therapies.

## 2 CHK1 Structure and Activation

The functional relevance of CHK1 is closely linked to its structural conservation, making it a protein that has remained largely unchanged from yeast to humans [14]. CHK1 was initially identified in yeast and was found to be a kinase that is essential for cell cycle arrest following DNA damage [15]. Subsequent identification of homologs in *Drosophila melanogaster*, *Xenopus laevis*, *Mus musculus*, and *Homo sapiens* [16,17] has enabled more detailed studies on the role of CHK1 in regulating the replication checkpoint, S-phase progression, and cell survival [5].

### 2.1 CHK1 Structure

**Figure 2:**
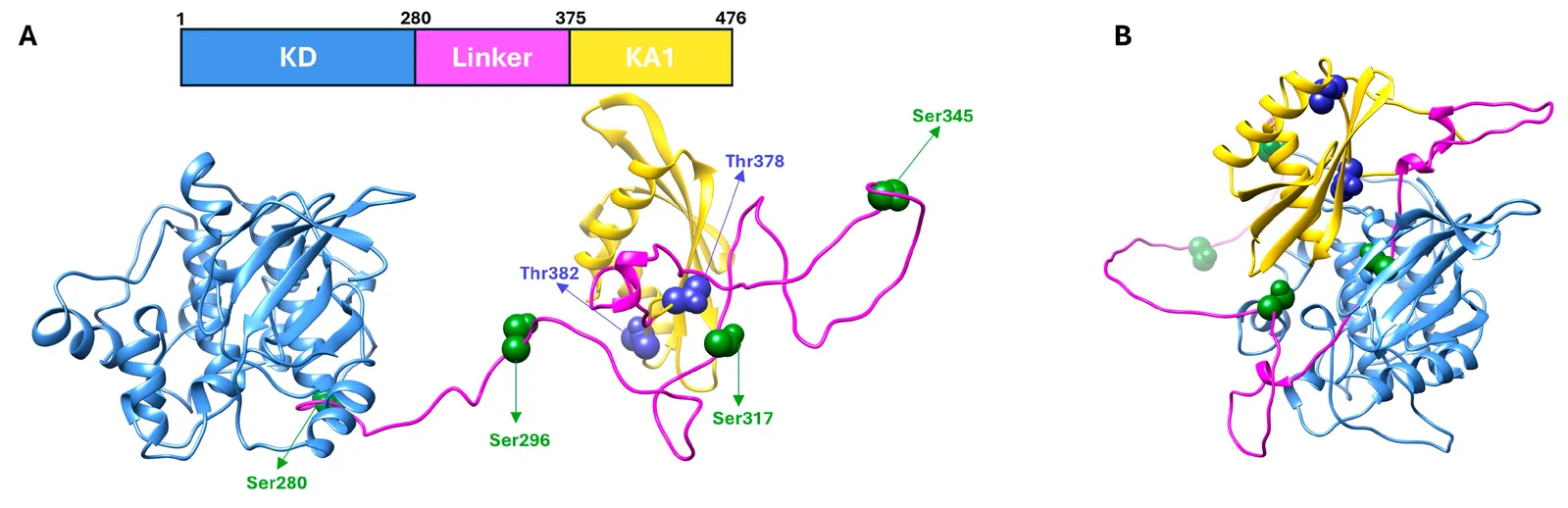
CHK1 protein structure. (**A**) Domain architecture (top) and open conformation (bottom) adopted by CHK1 following the phosphorylation of specific serine (in green) and threonine (in blue) residues. CHK1 consists of a kinase domain (KD, in blue, residues 1–280) and a KA1 domain (in yellow, residues 375–476), connected by a linker region (in pink, residues 280–375). (**B**) Closed and active conformation of CHK1 kinase, showing the interaction between the KD and KA1 domains. Coordinates obtained from UNIPROT database (https://www.uniprot.org/uniprotkb/O1 4757/entry#structure). Open conformation code: O14757_5-446:5iso.1.A; closed conformation code: AF-O14757-F1.

CHK1 belongs to the Ca^2+^/calmodulin-regulated Ser/Thr kinase family (CAMKK). It is a protein consisting of 476 amino acids with a molecular weight of 54 kDa [18,19]. All homologs are characterized by an *N*-terminal kinase domain (KD; residues 1–289) and a *C*-terminal kinase-associated regulatory region (KA1; residues 375–476), which are connected by a flexible linker region (residues 261–340) [20,21] (Fig. 2A).

The KD exhibits sequence homology and structural conservation across different species [20]. KD is characterized by a bilobal structure containing the ATP-binding site at its centre [20]. The two lobes of the KD are linked by β6′ of the hinge region and held together at the lobe interface by an extensive hydrogen-bond network involving β6′, the loop connecting αC and β4 of the *N*-terminal lobe, and strands β7 and β8 of the *C*-terminal lobe (Fig. 2A). The KD domain adopts an active conformation in which the ATP binding site, the catalytic residues, and the activation loop are well ordered [20,21]. In contrast to the activation loop of other kinases [22,23] the activation loop of KD does not require phosphorylation.

The KA1 regulatory domain was first described in yeast [24]. Despite the presence of significant structural homology, the degree of sequence identity among species is relatively low [18,25,26]. The conserved features of the KA1 domain include a four-stranded β-sheet flanked by two α-helices that form a concave hydrophobic core (βαββββα motif) [18,27] (Fig. 2A).

A physical interaction has been observed between KD and KA1 [28], suggesting a negative impact of KA1 on the kinase activity of KD. Indeed, isolated KD exhibits over 20-fold greater activity towards various substrates, such as Cdc25C, than the recombinant full-length CHK1 protein [20]. However, the absence of the KA1 region at the *C*-terminus results in a complete loss of CHK1 biological function. Indeed, in response to DNA damage KA1 positively regulates KD. This has been demonstrated by the fact that upon DNA damage-induced phosphorylation, intramolecular interactions between KD and KA1 are disrupted [18], thereby determining a further activation of CHK1 [20,25,27]. Interestingly, this phosphorylation involves residues located within the activation loop and requires KD activity. This suggests that the process of autophosphorylation plays a role in the regulation of CHK1 function [18,24,28,29,30,31,32] (Fig. 2B).

### 2.2 CHK1 Phosphorylation at Ser296, Ser317, and Ser345 during the DNA Damage Response

Ataxia telangiectasia and Rad3-related (ATR) kinase and CHK1 have been demonstrated to mediate cell cycle arrest in response to replication fork stalling or DNA damage [33,34,35,36]. During replication stress (RS) or single-stranded DNA (ssDNA) accumulation, the replication protein A (RPA) coats ssDNA, thereby facilitating ATR recruitment and activation via ATR-interacting protein (ATRIP) [19,37,38,39]. Once localized on the SSDNA, ATR phosphorylates RPA32 at Ser33, followed by cyclin-dependent kinases 1/2 (CDK1/2)-mediated phosphorylation at Ser23 and Ser29 [40]. The activation of ATR is further enhanced by topoisomerase 2-binding protein 1 (TOPBP1) and the RAD9-RAD1-HUS1 (9-1-1) complex [41,42,43]. Furthermore, the Ewing’s tumour-associated antigen 1 (ETAA1) binds to RPA and promotes ATR activation [44,45]. Additionally, the TIMELESS/TIPIN complex enhances the interaction between Clastin and RPA [46]. In the initial phase of the DDR, ATR also phosphorylates histone H2AX at Ser319, leading to the formation of γH2AX, a key marker of DNA damage [47].

The accumulation of the ATR/ATRIP complex is essential to induce the ATR-dependent CHK1 phosphorylation at Ser317 and Ser345, both of which are located within the *C*-terminal KA1 domain (Table 1) [19,21,39,48,49,50,51,52,53]. Phosphorylation at Ser317 is a critical regulatory event that is primarily mediated by the ATR kinase in response to RS and genotoxic damage. This post-translational modification plays an essential role in activating the CHK1 checkpoint functions, thereby preventing premature mitosis and ensuring proper cell cycle arrest and DNA repair [54]. Specifically, phosphorylation at Ser317 is required for the subsequent phosphorylation of other key residues, including Ser296 and Ser345 (Table 1). Collectively, there residues promote full CHK1 activation during the DDR. Notably, the mutation of Ser317 impairs checkpoint activation and replication fork progression yet does not necessarily affect cell survival in the absence of stress. This observation suggests that this site may have distinct functional roles in addition to DDR [54,55,56,57,58,59,60]. CHK1 phosphorylation at Ser345 facilitates its retention in the nucleus and enhances its stability. This phosphorylation is primarily mediated by the ATR kinase in response to RS and DNA damage and serves as the final and essential modification for full activation of the CHK1 checkpoint functions, including cell cycle arrest and DNA repair [19,61,62]. Indeed, CHK1 phosphorylated at Ser345 accumulates both in the nuclear pool and, more prominently, at chromatin regions containing RPA-coated ssDNA [19,61,63].

CHK1 can also be phosphorylated at Ser286 and Ser301 by CDK1 (Table 1) [54]. These post-translational modifications facilitate full CHK1 activation, ensuring proper checkpoint function [64]. Following the checkpoint signalling, the Ser345-phosphorylated CHK1 located within nucleoplasm undergoes ubiquitination and proteasomal degradation, a process which is mediated by CRL4–DDB1–CDT2. In contrast, the cytoplasmatic cytoplasmic CHK1 is degraded by CRL1–SKP1–Fbx6 [63].

### 2.3 CHK1 Phosphorylation at Ser280, Ser286, Ser301, Thr378, and Thr382

Although most studies on CHK1 activity focus on the phosphorylation of Ser296, Ser317, and Ser345, it is well established that the phosphorylation of additional residues is also critical for CHK1 regulation (Table 1) [19,49,52,56].

The Ser280 phosphorylation is catalysed by several kinases including the Akt serine/threonine kinase (AKT) [65,66], ribosomal s6 kinase (RSK) [67], proviral integration site for moloney murine leukaemia virus-1 (PIM1) [68], and AMP-activated protein kinase (AMPK) [69]. The functional outcome varies depending on the upstream kinase, cell type, and growth conditions [19]. For instance, during the G0/G1 transition, Ser280 is phosphorylated by the mitogen-activated protein kinase (MAPK)/MAPK-activated protein kinase-1 (also known as 90 kDa RSK or p90RSK) downstream of the Ras–MAPK cascade [39,67], facilitating CHK1 translocation from the cytoplasm to the nucleus and maintaining genome integrity during cell cycle progression [67]. Consequently, nuclear accumulation of the Ser280-phosphorylated CHK1 accelerates the subsequent phosphorylation at Ser296 and Ser345 CHK1 residues [67]. In contrast, AMPK-dependent phosphorylation occurs during glucose deprivation, promoting recognition by β-TrCP proteins, which mediate ubiquitination and degradation of CHK1 [69].

Further CHK1 residues that undergo phosphorylation are Ser286 and Ser301, which are targeted during mitosis by CDK1 [54]. These phosphorylation events promote CHK1 nuclear export at the end of G2 phase, thereby alleviating CHK1-mediated inhibition of CDK1 and supporting mitotic progression [39,70,71,72,73]. Interestingly, both CDK1 and CDK2 can phosphorylate Ser286 and Ser301 during the DDR, in addition to the ATR-dependent phosphorylation of Ser317 and Ser345. This finding suggests that proliferative and checkpoint signals may coexist [59,66,71].

It has been demonstrated that Thr378 and Thr382 are additional sites of CHK1 autophosphorylation [32]. These residues are located within the KA1 domain, in a conserved region associated with ubiquitination and proteasomal degradation [74]. Despite this localisation, the autophosphorylation of Thr378 and Thr382 is associated with both CHK1 activation and degradation. On the one hand, these two residues stabilize the kinase in an active conformation. Conversely, they unveil a region that is recognised by the CRL1–FBX6 complex, which targets the protein for proteasomal degradation [32].

**Table 1:** CHK1 phosphorylation sites and their functional roles.

Site	Stimulation	Kinase	Interactor	Effect on CHK1 Function	Ref.
*Ser280*	Growth factor	p90RSK		Transport from the cytoplasm to the nucleus	[67]
	UV irradiation	p90RSK		Acceleration of activation processes	[67]
*Ser286*	G2/M transition	CDK1		Transport from the nucleus to the cytoplasm	[39,70,71,72,73]
*Ser301*					
*Ser296*	DDR	Auto phosphorylation	14-3-3γ	- Interaction with Cdc25A via 14-3-3γ - Release from DNA damage sites within the nucleus	[72]
*Ser317*	DDR	ATR		Catalytic activation	[19]
*Ser345*					
*Ser345*	DDR	ATR	14-3-3γ	Nuclear retention	[19,63]
	DDR	ATR		PolyUb and degradation	[69]
*Thr378*	Intramolecular inhibition of KA1 domain	Auto phosphorylation		G2 cell cycle arrest Proteasomal degradation	[32]
*Thr382*					

Abb: ATR, Ataxia telangiectasia and Rad3 related; CDK1, cyclin-dependent kinase 1; DDR, DNA damage response; p90RSK, 90 kDa ribosomal s6 kinase; UV, ultra-violet.

## 3 CHK1 and Cell Cycle Regulation

The role of CHK1 in the transition from the G1 phase to the S phase has been recognised through the CHK1-dependent control of Cdc25A [75,76]. In engineered colon cancer cells, depletion of CHK1 induces cell cycle arrest at the S or G2 phase, but not at the G1 phase or the G1/S transition. CHK1 exerts its regulatory influence over the normal cell cycle primarily by inducing the turnover and subsequent degradation of Cdc25A [75,76]. CHK1 depletion in early mouse embryos determines Cdc25A degradation, influencing cell cycle transitions even in the absence of exogenous DNA damage [77]. Indeed, during the initial stages of embryo development, the duration of the G1 and G2 phases of the cell cycle must be modified for the correct duplication of the blastomeres. This molecular event deregulates CDK1 kinase activity, thus leading to a shortening of all phases of the cell cycle [77].

The Cdh1 protein plays a pivotal role in controlling cell division at the end of mitosis and in the subsequent G1 phase of the cell cycle. The regulation of this process by CHK1 at the G1-to-S phase transition in cancer cells has been demonstrated by the discovery that the anaphase-promoting complex/cyclosome (APC/c), a ubiquitin ligase involved in controlling diverse physiological processes, including cellular proliferation, is a CHK1 substrate. Cdh1 is indeed one of the co-activator proteins of APC/c and thus contributes to the regulation of protein degradation by providing substrate specificity to the E3 ligase in a cell-cycle-regulated manner. Phosphorylation of APC/c-Cdh1 allows its recognition by the SCFβ-TrCP ubiquitin ligase complex. The subsequent proteasomal degradation of APC/c-Cdh1 is required for cell cycle progression. In this context, CHK1 is required to phosphorylate APC/c-Cdh1 during the normal, undisturbed cell cycle, thereby activating the molecular mechanisms that lead to its degradation and determines S-phase entry and cell proliferation [78].

The essential regulatory role of CHK1 during mitotic progression has been demonstrated using mutants of the CHK1 phosphorylation sites, i.e., Ser317 and Ser345. The mutation of the Ser317 site was found to abrogate DNA replication and to block the G2/M transition, without affecting cell viability. Conversely, a mutation at the Ser345 site was lethal for cells. These disparities appear to be dependent on the different phosphorylation modes of these two residues, with Ser345 phosphorylation initiated at the centrosome during unperturbed mitosis, independently of CHK1 phosphorylation induced by DNA damage. These observations suggest that CHK1 plays a physiological role in mitosis that is independent of exogenous DNA damage [56]. CHK1 phosphorylation also occurs through CDK1 activation at Ser286 and Ser301 during mitosis. This process contributes to the sequestration of CHK1 in the cytoplasm, a prerequisite for the inhibition of CHK1 and the induction of mitotic entry. It is therefore evident that these molecular events ensure that CHK1 can regulate the proper timing of the G2/M transition in normal cell cycles [71].

CHK1 can also act during spindle checkpoint progression in the absence of DNA damage. Depletion of CHK1 in HeLa cells causes a loss of kinetochore function, triggering mitotic catastrophe by activating spindle checkpoint mechanisms and reducing polo-like kinase 1 (PLK1) activity. First, CHK1 is required for correct chromosome positioning before spindle checkpoint activation, thereby ensuring microtubule-kinetochore attachments. Subsequently, CHK1 inhibits PLK1 activity, thus enabling cells to progress through the spindle checkpoint and induce mitotic exit [79].

The non-canonical control of cell proliferation by CHK1 in the absence of DNA damage has recently been attributed to the regulation of cellular metabolism. This regulation occurs through the direct physical interaction of CHK1 with core metabolic enzymes that influence the glycolytic pathways and cellular energy utilisation [13,80]. Furthermore, it has been demonstrated that metabolic pathways have the capacity to influence the stability of CHK1. Indeed, glucose deprivation in cancer cells has been shown to induce AMPK activation that subsequently phosphorylates CHK1 [69]. This process drives CHK1 proteasomal degradation via the SCFβ-TrCP ubiquitin ligase complex, which tags CHK1 with ubiquitin chains, targeting it for destruction by the 26S proteasome [69]. The reduction in intracellular CHK1 levels results in reduced checkpoint activation, increased risk of RS, and heightened genomic instability, even in the absence of exogenous DNA damage [12,65,73,74,75,77]. This provides an important mechanism by which glucose metabolism regulates a DNA damage effector, and implies that glucose deprivation, which is a common hallmark of solid TME, enhances mutagenesis, clonal expansion, and tumour progression by triggering CHK1 degradation [69]. This proves that the proteolytic control of CHK1 is a critical aspect of cell cycle regulation and cancer biology.

## 4 CHK1 and Chromatin Modifications

Histone post-translational modifications have been shown to shape chromatin states, which in turn regulate transcription, DNA replication, and repair. Under physiological conditions, a significant proportion of CHK1 is associated with chromatin, even in the absence of DNA damage, suggesting a pivotal role of this kinase in non-canonical functions related to the regulation of chromatin structure and transcriptional activity [10,11]. Following genotoxic stress, the activation of ATR kinase and the subsequent phosphorylation of CHK1 (at key sites such as Ser317 and Ser345 residues) trigger the DDR and cause a profound reorganization of the CHK1 subcellular localisation, resulting in rapid dissociation from chromatin [10,11,55,81]. This dissociation is functionally significant as it restricts the ability of CHK1 to directly regulate the status of histone modifications at specific chromatin loci [11]. This remodulates the transcriptional programme, including the repression of genes involved in cell cycle progression, contributing to the integration of DDR and epigenetic control [82].

CHK1 is a histone 3 (H3) kinase, as it phosphorylates the Thr11 (H3T11) residue within the nucleosome [82,83]. In undamaged cells, CHK1 is associated with chromatin and phosphorylates H3T11 (H3T11ph), thereby promoting gene transcription (e.g., *cyclin B1* and *CDK1* genes) and cell proliferation [11,82]. Specifically, H3T11 phosphorylation promotes the recruitment of the lysine acetyltransferase 2A (KAT2A, also known as GCN5) to the promoters of cell cycle genes, thereby inducing the acetylation of the Lys9 residue of H3 (H3K9ac) [11,84,85,86]. Moreover, H3T11ph activates JMJDC2C (also known as KDM4C), a histone demethylase that removes methyl groups from H3 Lys9 (H3K9) [87]. Following DNA damage, the rapid dissociation of CHK1 from chromatin reduces H3T11ph, impairs the expression of cell cycle genes, and promotes cell cycle arrest [11,87].

CHK1 also phosphorylates serine residues of the H3 histone. The phosphorylated Ser10 residue (H3S10ph) blocks demethylation of the Lys9 residue (H3K9) by the histone demethylase JMJD2A [88]. Of note, CHK1 does not engage in T11 phosphorylation when Ser10 (H3S10) has already been phosphorylated [89]. Indeed, H3S10ph is detected during transcriptional activation in interphase and during chromosome condensation and segregation during mitosis [90], whereas H3T11ph is implicated in transcriptional control in interphase [11]. Therefore, dual CHK1-dependent phosphorylation on H3 may represent gene-specific chromatin signals in interphase that regulate transcriptional activation [89].

CHK1-dependent phosphorylation of the Ser57 residue (H3S57ph) affects nucleosome dynamics and influences RS response and DNA repair processes [91]. Mechanistically, the H3S57ph weakens DNA-histone interactions, enhances nucleosome mobility, and intersects with H3K56 functionally, slowing S-phase progression post-arrest [91].

In most somatic human cells, the CHK1-dependent phosphorylation of Ser31 of the histone variant H3.3 (H3.3S31ph) localizes specifically to pericentric satellite DNA repeats during mitosis. In contrast, alternative lengthening of telomeres (ALT) cancer cells exhibit high H3.3S31ph levels across entire chromosomes due to elevated CHK1 activity. Inhibiting CHK1 pharmacologically during mitosis or expressing the H3.3S31A mutant in ALT cells reduces H3.3S31ph and elevates γH2AX (H2AX histone phosphorylated at Ser139) on chromosome arms and telomeres. This mark maintains chromatin integrity, prevents γH2AX accumulation, and supports survival in ALT cancers [92]. Of note, CHK1 inhibition in these cancer cells decreases cell viability [92].

During the replication of heterochromatic regions under basal conditions, KAP1 phosphorylation at Ser473 triggers its interaction with several DNA replication factors, including PCNA, MCM3, and MCM6 [93]. The Suv39h1 KMT is then recruited, enabling the tri-methylation on Lys9 of newly incorporated histone H3 molecules, after they have been monomethylated by the CAF1–KAP1–HP1–SETDB1 complex [94]. The KAP1 phosphorylation at Ser473 is also required to promote H4K20me3 and H3K64me3 [93]. CHK1 phosphorylates KAP1 at Ser473 in physiological conditions [95]. In response to stalled replication fork induced by either HU or UV, CHK1-induced KAP1 phosphorylation led to an increased binding of KAP1 with E2F1, thereby reducing the ability of E2F1 to activate the expression of a subset of proapoptotic genes. The CHK1-dependent phosphorylation regulates the KAP1 transcriptional repression activity. Indeed, KAP1 acts as a co-repressor for more than 200 Kruppel-associated box zinc finger proteins (KRAB-ZFPs), the largest family of transcription factors in humans. KRAB-ZFPs first bind their target DNA sequences, recruiting KAP1 to form a scaffold complex with HP1, SETDB1, and histone deacetylases, thereby silencing genes through local heterochromatin formation at the locus [96]. The loss of Ser473 phosphorylation results in diminished BRCA1 focus formation, slower kinetics of DNA damage repair, and elevated rates of cell death subsequent to DNA damage. This highlights the key role of KAP1 Ser473 phosphorylation in DDR and cell survival [95].

## 5 CHK1 and Cell Differentiation

CHK1 plays a multifaceted regulatory role in cell differentiation. It prevents cell cycle exit associated with trophoblast stem cell differentiation by mediating the degradation of CDK inhibitors, such as p57 and p21, through phosphorylation [7]. This preserves the proliferative capacity of these progenitor cell populations. Conversely, CHK1 is essential for the proper differentiation of the T lymphoid lineage in thymocytes [97] and for haematopoietic stem cell maturation [98]. In this context, CHK1 promotes lineage commitment and balanced differentiation.

Experimental inhibition of CHK1 has demonstrated that disruption of its function can have detrimental effects on normal tissues. For instance, haematopoietic stem cells lacking CHK1 activity exhibit reduced myeloid precursor generation and abnormal skewing towards lymphoid differentiation, highlighting the vital role of CHK1 in maintaining haematopoietic integrity [99,100]. Furthermore, the differentiation of acute myeloid leukaemia (AML) cells using drugs such as cytarabine or *de novo* pyrimidine synthesis pathway inhibitors is critically dependent on CHK1 activation, demonstrating its contribution to physiological and therapeutically induced differentiation [101,102].

CHK1 has been shown to influence myeloid cell differentiation by modulating multiple molecular pathways and cellular mechanisms. Inhibition of CHK1 using MK-8776 leads to an upregulation of CHIP E3 ligase and proteasome 26S subunits [103], enhancing proteasome-mediated degradation of oncoproteins like Bcr-Abl and PML-RARα, which are implicated in leukemogenesis and the block of cell maturation [103,104]. These findings suggest that CHK1 activity is linked to the regulation of proteasome function and protein turnover that are fundamental for differentiation processes in myeloid cells [103,104]. Further, CHK1 inhibition by either MK-8776 or prexasertib activates caspase-3 and caspase-1 through non-apoptotic pathways, thereby supporting the concept that caspases serve roles beyond apoptosis, acting as critical mediators of cell differentiation and stem cell function. Moreover, CHK1 inhibition promotes the upregulation of IRF8, a transcription factor essential for both myeloid lineage maturation and induction of inflammatory cytokines, fortifying the link between DDR signalling, inflammation, and cellular differentiation [104]. CHK1 inhibition has been shown to impact epigenetic regulators such as KDM1A (LSD1), FOXM1, c-Myc, and SMARCB1, whose downregulation aligns with reduced proliferation and promotion of differentiation in AML cells. The upregulation of CDKN2A and activation of the p53 axis further contribute to cell cycle arrest, favouring maturation over proliferation [104].

Despite the apparent risks and limitations, new findings suggest that differentiation programmes initiated by DDR proteins, such as ATM [105], p53 [106], and p21 [107], may interact with CHK1-regulated pathways in complex ways. The influence of the combination of CHK1 inhibition and feedback activation of DNA damage on cancer cell differentiation in the absence of direct genotoxic stress remains to be clarified. These intricacies highlight the need for further research to evaluate the precise effects of iCHK1, which could optimise anti-cancer strategies by targeting aberrant differentiation while preserving normal tissue function.

As mentioned above, H3T11ph is a chromatin modification that contributes to the maintenance of an open and accessible euchromatic state, which is essential for the expression of key genes for mitotic progression, such as cyclin B1 and CDK1 [11,108]. This event ensures that stem cells and their precursors remain within an active proliferative pool, an essential requirement before they embark on the differentiation pathway [7]. This event plays an important role in the context of stem cells and their progeny, where the coordination of chromatin accessibility and transcriptional programs is essential for maintaining proliferative capacity prior to lineage commitment [8]. Although the specific role of CHK1-mediated H3T11ph in hematopoietic stem cells (HSCs) has not yet been fully characterized, CHK1 has been implicated in the control of differentiation programs and transcriptional regulation in hematopoietic progenitors [98]. Furthermore, epigenetic studies in HSCs demonstrate that active histone marks, including the H3K9ac, are associated with lineage-affiliated genes and dynamic regulation during myeloid differentiation [109]. In addition to its transcriptional scaffolding function, basal CHK1 activity also correlates with a safeguard mechanism that prevents premature exit from the cell cycle, which is typical of terminal differentiation [7]. By maintaining the acetylated state of promoters and stabilising key inhibitors such as p57 and p21, CHK1 ensures that the cell responds appropriately to differentiation stimuli [7,11]. Without this epigenetic control, stem cells lose the ability to correctly interpret environmental signals, resulting in incomplete or dysfunctional differentiation [7].

The complex network of epigenetic control mediated by CHK1 extends to the regulation of heterochromatin stability, involving direct interaction with the co-repressor KAP1 and the demethylase JMJD2C [82,93,110]. In undisturbed physiological contexts, CHK1 maintains constitutive levels of KAP1 phosphorylation, thereby modulating its affinity for chromatin and influencing its function as a platform for various remodelling enzymes [93,95]. JMJD2C plays a pivotal role in removing trimethylation residues from histone H3 lysine 9 (H3K9me3), a modification associated with gene silencing and the formation of facultative heterochromatin [111,112]. In the absence of genotoxic stress, this mechanism enables CHK1 to prevent the accumulation of H3K9me3, thereby ensuring that accidental chromatin compaction does not prematurely silence pluripotency or differentiation programmes [9]. The synergy between the H3T11ph/H3K9ac axis, which promotes chromatin opening, and the KAP1/JMJD2C axis, which prevents closure, defines a bidirectional control system [11], allows stem cells to preserve their transcriptional competence, and ensures that differentiation only occurs in response to appropriate physiological signals [11,98].

## 6 CHK1 and Cellular Metabolism

To generate two daughter cells, it is necessary for proliferating cells to double their biomass during the cell cycle. Ensuring an adequate supply of dNTPs for DNA synthesis and of ATP for cell division is essential for progression through S phase and mitosis. The role of CHK1 in genome maintenance is closely linked to these metabolic demands. In addition to monitoring genomic integrity, CHK1 coordinates the timing of DNA replication with the availability of metabolic resources. By doing so, CHK1 prevents unscheduled or excessive DNA synthesis that could overwhelm the cell’s metabolic capacity, thereby limiting RS and the accumulation of DNA damage. Insufficient nutrients or energy can trigger cell cycle checkpoints, particularly those regulated by CHK1. This results in the arrest of the cell cycle and prevents the disruption of metabolic and genetic processes [113,114,115].

### 6.1 CHK1 and Cell Proliferation

**Figure 3:**
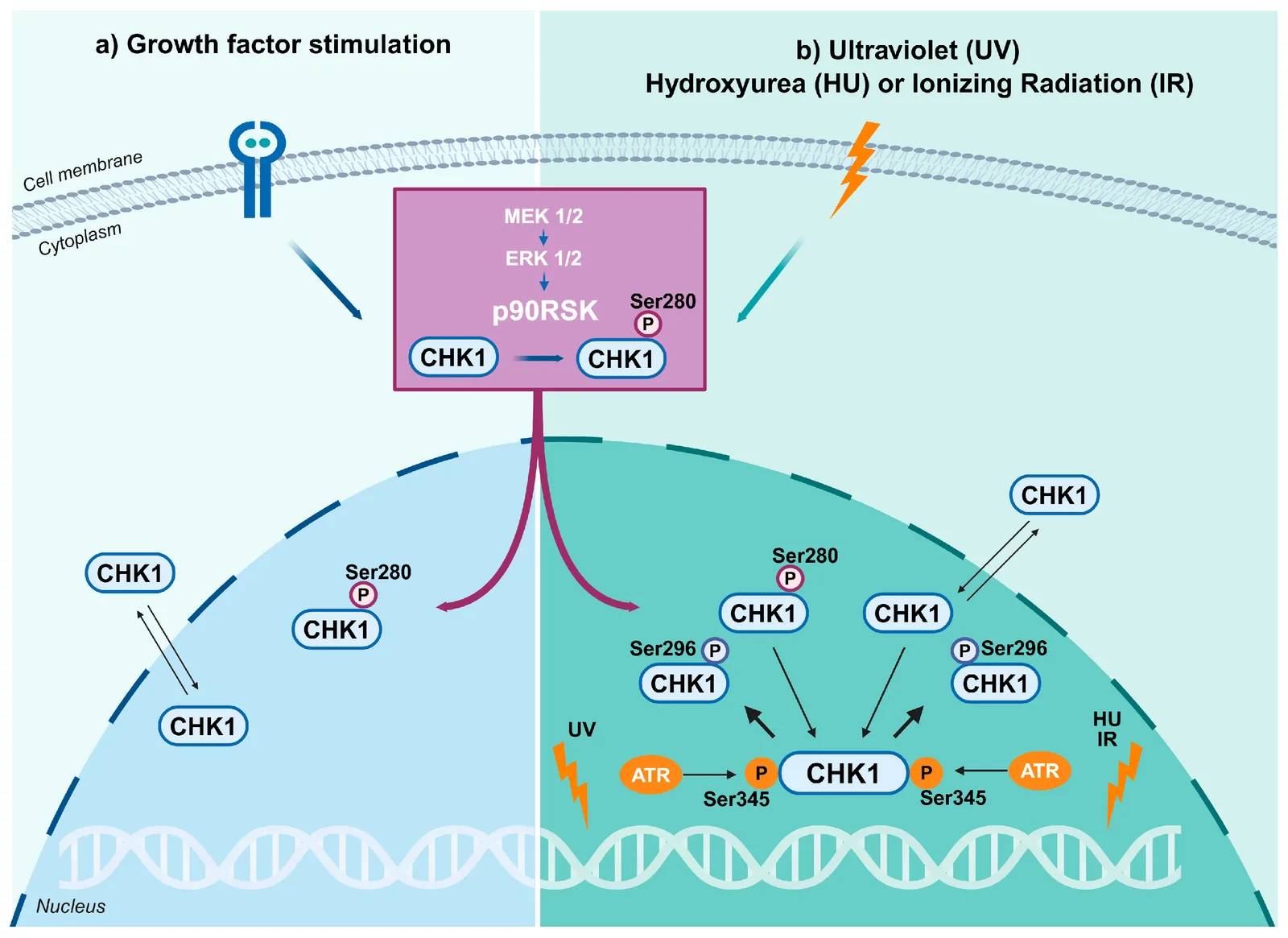
p90RSK controls the localization and activation of CHK1 through phosphorylation at Ser280. (**a**) Upon activation downstream of growth factor signalling, p90RSK phosphorylates CHK1 at Ser280, promoting its nuclear translocation and retention. (**b**) Exposure to DNA-damaging agents such as UV, HU, or IR triggers CHK1 phosphorylation on Ser345 by ATR and its autophosphorylation on Ser296. Meanwhile, increased phosphorylation of Ser280 by p90RSK enhances the kinase’s overall activity in the DNA damage response [67]. Created in https://BioRender.com.

CHK1 plays two seemingly opposing functions: cell cycle arrest and proliferation. These are highly context-dependent and modulated, at least in part, by the mitogen-activated protein kinase (MAPK)/p90RSK pathway. The MAPK/p90RSK acts downstream of the Ras pathway [13,67]. The process of cell proliferation depends upon the timely reception of extracellular growth factor signals, which are primarily transmitted through two core pathways downstream of receptor tyrosine kinases (RTKs). The phosphatidylinositol 3-kinase (PI3-K)/Akt/protein kinase B (PKB) pathway and the Ras/MAPK cascade, composed of Raf/MAPK kinase (MEK)1/2/extracellular signal-regulated kinase (ERK)1/2, are two such pathways [116,117]. p90RSK, a Ser/Thr kinase, acts downstream of this latter pathway. Upon growth factor stimulation, p90RSK is phosphorylated by ERK1/2 on multiple residues by several kinases and thereby activated. The activation of p90RSK results in the phosphorylation of CHK1 at Ser280, thereby promoting its nuclear retention [67], where it undergoes further phosphorylation at Ser296 and Ser345 [61,72,118] (Fig. 3). As outlined above, the phosphorylation of Ser296 and Ser345 residues is essential for the DDR. Interestingly, cell exposure to DNA-damaging agents such as ultraviolet (UV) light, ionising radiation (IR), and hydroxyurea (HU) also induces the p90RSK-dependent phosphorylation of CHK1 at Ser280, which is subsequently phosphorylated at Ser296 and Ser345 to promote the DDR [119,120]. This finding indicates a convergence between the ATR-CHK1 and p90RSK pathways, thereby facilitating nuclear CHK1 accumulation. This is imperative for preserving genomic stability during periods of cell proliferation. The Ras/MAPK/RSK axis is frequently found to be overexpressed in a variety of human cancers. Therefore, its interaction with CHK1 represents a pivotal nexus that connects oncogenic signalling with genome maintenance and metabolic regulation [119,120].

### 6.2 CHK1, Redox Balance, and Nucleotide Synthesis

CHK1 is an antioxidant regulator. Indeed, CHK1 is directly involved in nuclear reactive oxygen species (ROS) sensing, particularly H_2_O_2_ [121]. CHK1 senses nuclear H_2_O_2_ through the oxidation of the conserved Cys408 residue, which is located in KA1. Oxidation of Cys408 activates CHK1 kinase activity. Once activated, CHK1 phosphorylates the single-stranded DNA-binding protein (SSBP1) and prevents it from localising to the mitochondria. This CHK1-dependent pathway is pivotal for regulating the flow of signals between the nucleus and the mitochondria. It also plays a key role in reducing nuclear ROS levels, thereby contributing to the maintenance of cellular redox homeostasis [121]. Importantly, this pathway contributes to platinum-based chemotherapies’ resistance in ovarian cancer models. Importantly, this links CHK1-mediated ROS sensing and regulation to chemoresistance mechanisms [121].

CHK1 also maintains dNTP homeostasis and supports the function of ribonucleotide reductase (RNR), which is the rate-limiting enzyme in dNTP synthesis under cellular redox stress [122]. RNR tightly controls the size of the dNTP pool, and disruption to its enzymatic activity results in dNTP insufficiency, RS, and S-phase accumulation [123,124,125]. Mammalian RNR consists of two homodimers, RRM1 and RRM2. RRM1 levels remain relatively constant throughout the cell cycle, but RRM2 accumulates during S phase and declines as cells progress into mitosis [126]. RNR activity requires electron transfer from both internal and external redox systems to sustain the reduction of ribonucleotides to deoxyribonucleotides. Disruption to this process impairs redox cycling, depletes dNTP pools, and exacerbates RS [127]. During RS, transcription of RRM2 is induced in an E2F1-dependent manner downstream of the ATR/CHK1 pathway [128,129]. Furthermore, UV-induced DNA damage relocates RRM1 and RRM2 from the cytoplasm to the nucleus, a process that depends on ATR/CHK1 signalling [122]. Depletion or inhibition of the thioredoxin (Trx) system, which normally keeps RNR subunits in a reduced functional state [130,131], enhances RRM1 oxidation. This slows replication fork progression due to an imbalance in dNTPs. In this context, CHK1 is activated downstream of ATR, leading to E2F1-dependent upregulation of RRM2 (i.e., the regulatory subunit of RNR). This compensates for the dNTP insufficiency caused by RRM1 oxidation [122]. CHK1 signalling also promotes the nuclear relocation of the two RNR components following DNA damage, thereby ensuring continued dNTP synthesis under stressful conditions. This mechanism supports DNA replication and genome stability, even in the face of oxidative conditions that threaten replication fork progression. Inhibiting CHK1 in Trx1-depleted contexts leads to reduced RRM2 expression, decreased tyrosyl radical activity, and limited RRM1 oxidation, which in turn impairs replication. CHK1 is therefore vital for safeguarding against RS by maintaining an equilibrium in the levels of dNTPs, facilitating their synthesis, and regulating the activity of redox-sensitive proteins within the nucleus. This makes it an indirect yet pivotal antioxidant regulator in situations of genotoxic and oxidative stress [122].

### 6.3 CHK1 and Glycolysis

**Figure 4:**
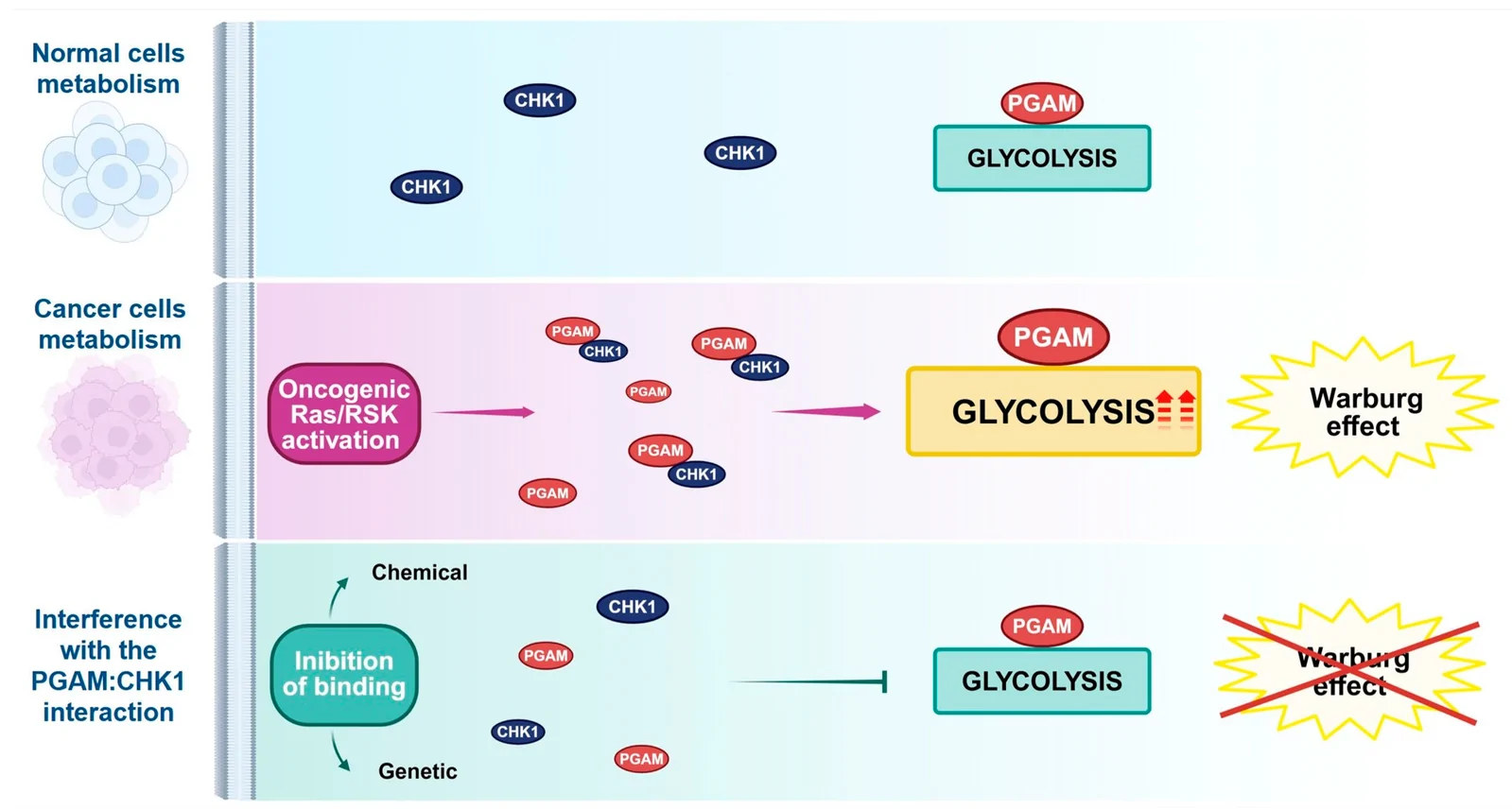
The interaction between PGAM and CHK1 contributes to the potentiation of glycolysis in cancer cells. Overexpression of PGAM in tumour cells, alongside activation of the Ras/RSK oncogenic pathway, promotes interaction and cooperation with CHK1. This leads to dysregulation of the glycolytic process and to the enhancement of the Warburg effect, which plays a key role in tumour progression. The genetic or pharmacological inhibition of CHK1:PGAM interaction interferes with glycolysis in cancer cells, thereby hindering their proliferation. Created in https://BioRender.com.

Oncogenic Ras/RSK activation is closely associated with the interaction of CHK1 with phosphoglycerate mutase 1 (PGAM1), a glycolytic enzyme that catalyses the interconversion of 3-phosphoglycerate (3-PG) to 2-phosphoglycerate (2-PG) [132,133]. Beyond its canonical enzymatic role, PGAM1 contributes to physiological homeostasis as it undergoes diverse post-translational modifications (e.g., phosphorylation, acetylation, and ubiquitination) in response to environmental cues and stress [134,135,136]. In cancer cells, PGAM1 enhances glycolytic flux through its interaction with CHK1, particularly under conditions of oncogenic Ras activation. CHK1 binds PGAM1, thereby stabilising it enzymatically and transcriptionally, resulting in an increase in the expression of glycolytic genes (e.g., hexokinase 2, glucose-6-phosphatase isomerase, aldolase A, and pyruvate kinase). This increase in glycolytic activity and lactate production, in turn, creates an immunosuppressive acidic TME that is essential for the Warburg effect that accompanies cancerous proliferation [133] (Fig. 4). Of note, PGAM1 activity also modulates mitochondrial function [137,138] and the pentose phosphate pathway (PPP) [139,140,141]. Indeed, 3-BP, the substrate of PGAM1, can act as an inhibitor of the 6-phosphoglycerate dehydrogenase (G6PD), thereby reducing the nucleotide synthesis reaction in the PPP [142,143]. Indeed, the PPP plays a pivotal role in the cellular response to ROS by generating NADPH, which in turn supports antioxidant defences. This implies that the interaction between CHK1 and PGAM1 may also be involved in the defence against oxidative stress (see paragraph 6.2) [106,107,108].

CHK1 physically interacts with pyruvate kinase isoform M2 (PKM2), the rate-limiting enzyme in glycolysis that converts phosphoenolpyruvate to pyruvate while generating ATP. In cancer cells, PKM2 is predominantly present in a low-activity dimeric form that promotes the Warburg effect. This configuration diverts upstream glycolytic intermediates to biosynthetic pathways such as the PPP for NADPH/ribose production. This supports nucleotide synthesis, maintains redox balance against ROS, and facilitates rapid proliferation [144]. In cardiomyocytes, the interaction between CHK1 and PKM2 triggers PKM2 phosphorylation, thereby stimulating glycolysis and the PKM2-mediated transcription of genes involved in proliferative processes [80]. Despite the absence of quantifiable data concerning the impact of CHK1 on PKM2 interaction on the progression of the cell cycle through the various phases, it has been documented that CHK1:PKM2 interaction induces progression from G1 to S phase [80].

### 6.4 CHK1 and Oxidative Phosphorylation

Acetylation, a prevalent post-translational modification of mitochondrial proteins, frequently results in the loss of protein function [145,146]. The acetylation of mitochondrial proteins is primarily governed by sirtuin family members, particularly SIRT3 [147]. SIRT3 has been identified as a pivotal modulator of mitochondrial redox and inflammation, operating as a primary mitochondrial deacetylase [148,149]. This is due to the fact that SIRT3 is responsible for the deacetylation of some subunits of the respiratory chain complex I. It is noteworthy that SIRT3 is transcriptionally activated by NF-κB and relocates to mitochondria through the phosphorylation by CDK1 [150]. In the context of cardiomyocytes, CHK1 has been observed to phosphorylate SIRT3, thereby activating its deacetylase function. The inhibition of CHK1 by AZD7762 results in a decrease in the acetylation levels of the respiratory chain I subunits, owing to a concomitant decrease in the activated SIRT3 [12]. Consequently, this results in a disruption of the respiratory chain [12]. This highlights the CHK1-dependent cross-talk between mitochondrial protein acetylation and bioenergetics [146,148].

## 7 Role of CHK1 in Immunity

Under physiological conditions, the immune system recognises and eliminates transformed cells through a process known as immune surveillance. However, during tumorigenesis, neoplastic cells acquire the ability to remodel the TME and re-educate infiltrating immune cells, converting the immune response from anti-tumour to pro-tumour. The progression of many types of cancer is influenced by the TME and by the immune cells present within it [151,152].

**Figure 5:**
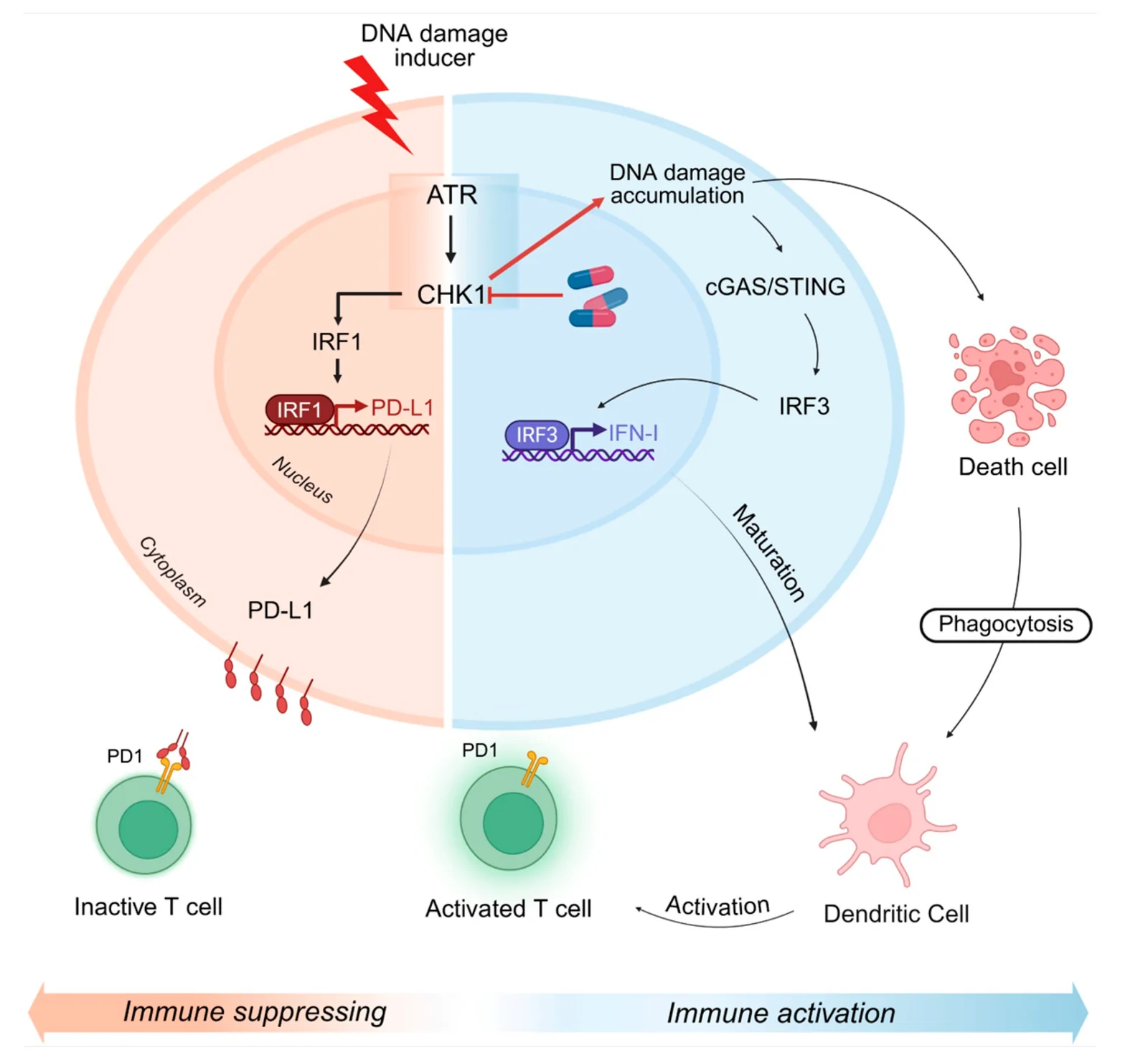
Role of CHK1 in the immune response. Under physiological conditions, DNA damage activates the ATR/CHK1 axis, which promotes IRF-1-dependent transcription of PD-L1. The PD-L1 protein localizes on the cell membrane, where it suppresses T cell activity by interacting with PD-1 expressed on T cell surface. However, therapeutic CHK1 inhibition promotes DNA damage accumulation and triggers the cGAS/STING pathway, driving IRF3 nuclear translocation, the transcriptional induction of IFN-I, and the subsequent maturation of dendritic cells (DCs). Mature DCs enhance T cell activation and potentiate the immune response. Persistent DNA damage can also induce immunogenic cell death, further amplifying immune activation via DC-mediated tumour cell phagocytosis. Created in https://BioRender.com.

Immunogenic cell death (ICD) is a specialised process of cell death characterised by the emission of stress signals known as danger-associated molecular patterns (DAMPs). ICD is also characterised by the release of ATP, the production of interferon (IFN) molecules, and the secretion of pro-inflammatory cytokines. Antigen-presenting cells (APCs), including dendritic cells, sense these signals and activate a cytotoxic T cell-mediated immune response. This leads to the effective elimination of cancer cells and promotes the development of long-lasting tumour antigen-specific immune memory [153,154,155,156,157] (Fig. 5). ICD is a major mechanism through which anticancer immunotherapies can stimulate the host immune system to attack cancer cells. Moreover, ICD shapes the immune landscape within tumours by promoting immune surveillance and long-lasting antitumour immunity [158].

ICD and DDR are tightly linked processes in the context of cancer biology and therapy. Many anticancer treatments induce DNA damage that, in turns activates the DDR. A change in the equilibrium between the formation and repair of DNA damage, caused by exposure to high levels of DNA-damaging agents or impaired DNA repair mechanisms, leads to the exposure and release of DAMPs and triggers the immune system [159,160,161]. This supports the notion that the DDR renders tumour cells more immunogenic.

Disruption to the immune balance and prolonged acute inflammation also stimulate the DDR network by causing DNA damage [162,163,164,165]. During inflammatory diseases such as neurological conditions, autoimmune disorders, and certain cancers, epithelial and inflammatory cells produce ROS and RNS. This leads to the formation of oxidative and nitrative DNA damage, which can inhibit key DNA repair proteins [166]. These forms of DNA damage can induce mutations and are thought to play a role in the initiation and/or promotion of inflammation-induced carcinogenesis [166].

### 7.1 CHK1 and the Immune Response

CHK1 plays a multifaceted role in tumour immunity, including enhancing immune surveillance and modulating immune cell infiltration and immune escape mechanisms within the TME. CHK1 is a key player in immune evasion mechanisms. Its increased expression in numerous types of cancer is clearly linked to the infiltration of various immune cells, including B cells, CD4^+^ and CD8^+^ T cells, macrophages, neutrophils, and dendritic cells, in several types of cancers [167]. This allows tumour cells to evade immune surveillance and resist immune-based therapies. Tumour cells use high CHK1 activity for the following reasons: (i) minimize exposure to or presentation of immunogenic tumour antigens, making them less visible to the immune system; (ii) promote TME acidification and consequently modulate infiltration and function of immune cells, decreasing recruitment or suppressing activity of cytotoxic lymphocytes; and (iii) promote expression of immunosuppressive molecules (e.g., PD-L1) on cancer cell surfaces, thereby inhibiting effective T-cell responses [1,167,168,169,170,171]. Accordingly, meta-analyses link high CHK1 expression to poor prognosis and low immune infiltration (e.g., reduced T-cells in pan-cancer) [172].

CHK1 inhibition enhances antitumor immunity primarily via DDR-dependent mechanisms. The pharmacological inhibition of CHK1 promotes cancer immune evasion reversal primarily through the DNA damage accumulation, which leads to cytosolic DNA release that in turn activates the cyclic GMP-AMP synthase-stimulator of IFN genes (cGAS-STING) pathway [173]. Activation of the cGAS-STING pathway leads to downstream signalling resulting in the phosphorylation and activation of the IFN regulatory factor 3 (IRF3), a key transcription factor that drives the expression of IFNs and inflammatory cytokines [174,175] (Fig. 5). The cGAS-STING pathway serves as a central regulator of DDR inhibitors driven immune activation, promoting Signal Transducer and Activator of Transcription 1/TNF-Related Apoptosis Inducing Ligand (STAT1/TRAIL)-dependent signalling and the direct stimulation of anti-tumour immune effector cells [173] (Fig. 5). Recent evidence indicates that, beyond the canonical immune signalling function, cGAS-STING and IRF3 also modulate DNA repair, checkpoint activation, and cell death [176,177]. Of note, the combination of radiotherapy and CHK1 inhibition seems to represent a therapeutic strategy for immunotherapy in ARID1A-deficient tumours, overcoming the current challenges associated with immune checkpoint blockers in colorectal cancer patients [172].

iCHK1 combined with HU (a well-known RS inducer) promotes pro-inflammatory cytokines and chemokines expression. Moreover, it induces immunogenic cell death that in turn promotes an adaptive anti-cancer cytotoxic response [178,179,180,181,182]. Notably, CHK1 inhibition activates caspase-1 via non-apoptotic pathways [104]. Caspase-1, a protease activated by inflammation, plays a pivotal role in processing and releasing key pro-inflammatory cytokines such as interleukin (IL)-1β and IL-18 [183], as well as activating inflammasomes [184,185]. Following CHK1 inhibition with MK-8776, transcriptomic analysis revealed significant upregulation of the IL1B transcript, highlighting the activation of inflammatory pathways and immune signalling [104]. MK-8776 also modulates a variety of other pathways that are important for the inflammatory response, such as the TREM1 and IL-17A axes, NRF-2-mediated oxidative stress, IL-8, neuroinflammation, HIF-1α-dependent signalling, and ferroptosis [104]. These changes confirm the immunomodulatory potential of iCHK1, which enhances cytokine and chemokine expression via classical mediators such as nuclear factor-κB (NF-κB) and cytosolic DNA sensors like the cGAS-STING pathway [104,186,187].

CHK1 inhibition enhances immunity also via DDR-independent inflammatory signalling. As previously reported, CHK1 promotes glycolysis and lactate production in cancer cells by binding to PGAM1 and PKM2, thereby enhancing glycolytic flux and supporting the Warburg effect. This leads to elevated extracellular lactate secretion, which dissociates into lactic acid and lowers the pH in the TME to acidic levels (pH 6.5–6.8). Acidic conditions suppress CD8^+^ T cell cytotoxicity and IFN gamma (IFNγ) signatures IFNγ signalling. Indeed, upon internalization of lactic acid through the SMCT2 transporter, CD4^+^ helper T cells and CD8^+^ cytotoxic T cells undergo phosphofructokinase inhibition and hexokinase downregulation, resulting in the inhibition of glycolysis and in the reduction of cell motility. In turn, T cells lose their responsiveness to chemokines and no longer migrate throughout the body [188]. This results in the inhibition of antitumor immune responses and the activation of potent, negative regulators of innate and adaptive immune cells [189]. Pharmacological CHK1 inhibition disrupts this process by blocking CHK1:PGAM1 interaction in cancer cells, reducing lactate efflux, normalizing pH, and restoring T-cell function via decreased PD-L1 on tumours and enhanced effector infiltration [190].

### 7.2 CHK1 and the PD-1/PD-L1 Immune Checkpoint

Immune checkpoints are essential immunoregulatory molecules that regulate the immune system, helping to maintain balance and prevent inappropriate immune responses under normal conditions. The Programmed cell death-1 (PD-1)/Programmed cell death-ligand 1 (PD-L1) axis is a fundamental immune checkpoint pathway that facilitates tumour immune evasion and represents a target for therapeutic intervention in cancer immunotherapy. PD-1 is an immune receptor that is expressed on activated CD4^+^ T cells and CD8^+^ T cells as well as on B cells in the periphery [191,192,193,194]. When PD-1 binds to PD-L1, it effectively blocks the immune escape of cancer cells by halting T cell proliferation and IFNγ production [191,195]. CHK1 plays a crucial role in regulating PD-L1 expression in tumour cells through its involvement in the DDR. Indeed, CHK1 regulates the transcriptional expression of PD-L1 by the canonical IRF1/STAT1/3 transcriptional pathway, and ATR stabilizes PD-L1 in a proteasome-dependent manner [155,196,197] (Fig. 5). This mechanism allows tumour cells to evade immune detection by increasing PD-L1 levels, which suppresses cytotoxic T-cell activity. Importantly, inhibiting CHK1 disrupts this pathway, reducing PD-L1 expression and potentially enhancing the efficacy of immune checkpoint therapies targeting the PD-1/PD-L1 axis. Thus, CHK1 represents a promising target for combination cancer therapies, thanks to its capability to link DDR to immune evasion by modulating PD-L1 expression. This makes it clear that CHK1 is key to overcoming tumour immune resistance [155,197,198].

The inhibition of CHK1 results in the accumulation of DNA damage in tumour cells, which in turn promotes the expression of more immunogenic antigens. This enhanced immune recognition can be achieved by increasing the presentation of tumour antigens or changing the expression of immunosuppressive molecules on the surface of tumour cells [169]. Combining iCHK1 with immune checkpoint inhibitors (such as PD-1/PD-L1 antagonists) has been shown to significantly boost anti-tumour immune responses. This is likely because CHK1 inhibition increases DNA damage and enhances immunogenicity in cancer cells [199]. Such enhanced immune recognition can be achieved by increasing the presentation of tumour antigens or changing the expression of immunosuppressive molecules on the surface of tumour cells. This dual strategy aims to maximize therapeutic efficacy by simultaneously targeting cancer cells through two complementary mechanisms [169].

## 8 Therapeutic Targeting of CHK1

iCHK1 have been investigated in various clinical settings, both as monotherapy and in combination with other anticancer agents, with mixed outcomes (Table 2).

### 8.1 CHK1 Inhibitors in Clinical Development

#### 8.1.1 Prexasertib (LY2606368)

Prexasertib (LY2606368) is a potent dual CHK1/CHK2 inhibitor. There is strong nonclinical evidence supporting its role in inducing replication catastrophe and sensitising tumour cells to DNA damage [200,201,202].

As a monotherapy, a Phase II single-arm pilot trial in BRCA wild-type triple-negative breast cancer (TNBC) demonstrated a modest overall response rate (ORR) of approximately 11%, though treatment was frequently associated with neutropenia and other hematologic toxicities [203]. In a separate Phase II trial, prexasertib also showed clinical activity in patients with recurrent BRCA wild-type high-grade serous ovarian carcinoma [202]. A Phase I trial was conducted in children with recurrent or refractory solid tumours, including central nervous system tumours. This trial established the maximum tolerated dose (MTD) and recommended Phase II dose (RP2D) [204].

Combination strategies with prexasertib have been explored, including DNA-damaging agents (e.g., cisplatin and cetuximab), and radiotherapy, particularly in the context of head and neck cancers [205]. Reflecting its therapeutic promise [202,206,207,208], prexasertib has received Fast Track Designation by the U.S. Food and Drug Administration for use as a monotherapy in certain ovarian and endometrial cancers.

#### 8.1.2 SRA737

SRA737 is an orally bioavailable iCHK1. It has been developed with a particular focus on combination strategies, especially with low-dose gemcitabine, a regimen that may result in reduced myelotoxicity compared to the use of full-dose gemcitabine alongside CHK1 inhibition [209].

SRA737 was evaluated in a Phase I/II open-label dose-escalation study as a monotherapy. This established an RP2D of approximately 800 mg once daily, with an MTD around 70 mg once daily. The reported toxicities included gastrointestinal effects such as diarrhoea and nausea/vomiting, as well as haematological adverse events, including neutropenia and thrombocytopenia [210].

In combination trials, the pairing of SRA737 with low-dose gemcitabine in a Phase I/II study yielded an ORR of approximately 10.8%, with improved tolerability. Notably, higher response rates were observed in certain cancer subtypes, including anogenital, cervical, high-grade serous ovarian, rectal, and small cell lung cancers [209].

Further trials are currently underway or scheduled in the future, encompassing combinations with agents such as the PARP inhibitor niraparib [210,211].

#### 8.1.3 GDC-0575

GDC-0575 is a highly selective small-molecule iCHK1 that has been investigated both as a monotherapy and in combination with gemcitabine [212].

In a Phase I dose-escalation trial, GDC-0575 was tested alone and alongside gemcitabine in patients with refractory solid tumours to evaluate its safety, tolerability, pharmacokinetics (PK), and to determine the MTD [212]. Beyond its cytotoxic potential, preclinical and animal model studies have demonstrated that GDC-0575 may also modulate the TME and inflammatory responses, such as colitis and colitis-associated cancer, through its effects on macrophage infiltration and cytokine expression. Whilst awaiting clinical validation, these findings indicate the potential of GDC-0575 to possess supplementary immune- or inflammation-modulatory characteristics [213]. Reported data from clinical trials demonstrate that clinical development of iCHK1 has progressed through both monotherapy and combination approaches, each informed by the mechanistic role of CHK1 in RS and cell cycle regulation.

### 8.2 Potential Combinatorial Therapies of CHK1 Inhibitors with Drugs Targeting Metabolic Pathways

The concomitant targeting of two parallel pathways regulating cell proliferation can result in a synergistic effect of the administered drugs [214]. In the case of iCHK1, their impact on pathways involved in DNA damage control is well established. Moreover, the evidence presented in this review highlights iCHK1’s influence on several metabolic pathways, suggesting its potential use in combination with metabolic pathway inhibitors. This approach would be particularly important given the high toxicity of iCHK1 in humans [215,216]. Therefore, considering the results of the clinical trials on iCHK1 outlined above, it is strongly recommended that these drugs be combined with agents that directly or indirectly target mechanisms regulating cell proliferation, in a manner analogous to those controlled by CHK1. This approach is essential to ensure that iCHK1 treatment doses can be reduced, thereby minimising adverse side effects in patients, specifically those of a haematological nature [215].

#### Preclinical Evidence Supporting CHK1 Inhibition Combined with Metabolic Targeting

Combination strategies that simultaneously target CHK1 and specific proteins involved in different metabolic pathways have shown strong synergy in multiple tumour types.

iCHK1 can be combined with inhibitors of *de novo* pyrimidine synthesis, particularly targeting dihydroorotate dehydrogenase (DHODH). In TNBC cell lines, the DHODH inhibitor teriflunomide synergized with CHK1 inhibition to trigger replication fork stalling, γH2AX accumulation, and apoptotic cell death. Mechanistically, nucleotide depletion induced by DHODH inhibition increases RS, while CHK1 inhibition disables the compensatory checkpoint response, accelerating lethal DNA damage accumulation [217].

**Table 2:** Clinical trials on prexasertib, SRA737, and GDC-0575 CHK1 inhibitors. Information has been extracted, summarized and modified from [218].

*CHK1 Inhibitor*	Trial ID	Trial Status	Phase	Combined	Cancer	Adverse Side Effects
***Prexasertib***	NCT02778126	C	I	-	Advanced cancer	Neutropenia, thrombocytopenia, fatigue, nausea, anemia
	NCT02735980	C	II	-	Small cell lung cancer	-
	NCT03414047	C	II	-	Ovarian cancer	-
	NCT02514603	C	I	-	Neoplasm	-
	NCT02873975	C	II	-	Advanced cancer	-
	NCT01115790	C	I	-	Advanced carcinoma	-
	NCT02203513	C	II	-	Ovarian, breast, prostate cancer	-
	NCT02808650	C	I	-	Refractory and recurrent childhood solid tumors and primary CNS	-
	NCT02860780	C	I	Ralimetinib	Advanced, metastatic, colorectal, non-small cell cancer	-
	NCT03495323	C	I	LY3023414	Cancer	-
	NCT03057145	C	I	Olaparib	Solid tumor	-
	NCT04095221	C	I/II	Irinotecan	Desmoplastic small round cell tumor, rhabdomyosarcoma	-
	NCT03735446	C	I	Mitoxantrone Etoposide Cytarabine	Acute myeloid leukemia, myelodysplastic syndromes	-
	NCT06597565	R	II	Gemcitabine	Head and neck squamous cell carcinoma	-
	NCT05548296	R	I/II	Gemcitabine	Platinum-resistant ovarian cancer, endometrial adenocarcinoma, urothelial carcinoma	-
	NCT04032080	C	II	LY3023414	Triple negative breast cancer	-
	NCT04023669	C	I	Cyclophosphamide Gemcitabine	Pediatric and molecular subtypes of medulloblastoma	-
	NCT02555644	C	I	Cisplatin Cetuximab	Head and neck neoplasm	-
	NCT02649764	C	I	Cytarabine Fludarabine Phosphate	Advanced myeloid neoplasms	-
	NCT02124148	C	I	Cisplatin Cetuximab Pemetrexed Fluorouracil LY3023414 Leucovorin	Neoplasm metastasis, colorectal neoplasms	-
***SRA737***	NCT02797964	C	I/II	-	Advanced solid tumors, NonHodgkin’s lymphoma	Nausea, vomiting, fatigue, diarrhea, anemia
	NCT02797977	C	I/II	Gemcitabine GDC-0575	Advance solid tumor	
***GDC-0575***	NCT01564251	C	I	Gemcitabine	Lymphoma, solid tumor	Neutropenia, anemia, nausea, fatigue, thrombocy

Combining iCHK1 with 5-fluorouracil (5-FU) enhances chemotherapeutic efficacy in colorectal cancer (CRC). 5-Fluorouracil (5-FU) is a pyrimidine analogue that exerts its cytotoxic effects primarily by interfering with nucleotide metabolism. Inhibition of CHK1 has been shown to sensitize cancer cells to 5-FU by disrupting S-phase arrest and impairing DDR pathways. For example, the iCHK1 NSC30049 potentiates 5-FU-mediated growth inhibition in CRC cells, including 5-FU-resistant lines, suggesting a role in overcoming chemoresistance [219]. Similarly, CHK1 knockdown via siRNA increased apoptosis in adenomatous polyposis colon (APC)-mutant CRC cells treated with 5-FU [220]. In p53-deficient CRC models, CHK1 inhibition with SB218078 enhanced 5-FU cytotoxicity, although complete restoration of sensitivity in resistant cells was not achieved, indicating the complexity of resistance mechanisms [221]. Moreover, targeting CHK1 in CRC stem cells augmented 5-FU cytotoxicity and reduced cell growth, supporting the potential of this strategy to eliminate resistant cell populations [222].

Several studies have demonstrated that combining iCHK1 with gemcitabine, a nucleoside analogue that exerts its cytotoxic effects by interfering with DNA synthesis, enhances DNA damage and apoptosis in multiple cancer models. For example, in pancreatic cancer cell lines, the iCHK1 LY2603618 potentiates gemcitabine-induced cytotoxicity by preventing S-phase checkpoint activation [223]. Similarly, in Ewing sarcoma, CHK1 inhibition increased gemcitabine-induced apoptosis both *in vitro* and *in vivo* [224]. In bladder cancer cells, the combination of gemcitabine and MK-8776 enhanced DNA damage, evidenced by γH2AX accumulation and poly(ADP-ribose) polymerase (PARP) cleavage [225].

CHK1 inhibition is also connected with NAD^+^/poly(ADP-ribose) (PAR) metabolism. Indeed, NAD^+^ functions not only as a central redox cofactor but also as an essential substrate for PARPs, thus creating a direct link between cellular metabolism and DNA repair. Upon sensing DNA damage, PARP1 and PARP2 catalyse the transfer of ADP-ribose units from NAD^+^ to target proteins, generating PAR chains that facilitate chromatin remodelling and recruitment of DNA repair machinery [226,227]. This PARylation consumes NAD^+^, producing nicotinamide as a byproduct, thereby connecting PAR metabolism to cellular NAD^+^ pools [228]. Hyperactivation of PARPs in response to extensive DNA damage can dramatically deplete NAD^+^, leading to impaired ATP production and to the activation of a regulated form of cell death known as parthanatos [228]. Conversely, the availability of NAD+ has been shown to modulate PARP activity, whereas NAD^+^ limitation restrains PARP-mediated signalling [229,230]. In ovarian cancer models, prexasertib has been combined with inhibitors of poly(ADP-ribose) glycohydrolase (PARG), which removes PAR chains from proteins and plays a crucial role in DNA repair by reversing the action of PARP enzymes [231], to potentiate cytotoxicity by driving unsustainable PARylation and NAD^+^ consumption. PARG inhibition traps PAR chains on chromatin and, when combined with CHK1 inhibition, leads to catastrophic RS, mitotic collapse, and loss of cell viability even in chemoresistant cancer cells [232].

Combinations of iCHK1 with inhibition of the PI3K/mTOR signalling axis have also demonstrated encouraging results. The dual PI3K/mTOR inhibitor samotolisib combined with prexasertib exhibited significant antiproliferative effects in TNBC cell lines [233]. Mechanistically, PI3K/mTOR inhibition suppresses anabolic metabolism and nutrient-sensing pathways, thereby depriving cells of the metabolic intermediates necessary for DNA replication. When combined with iCHK1, this metabolic stress becomes incompatible with cell survival, leading to profound replication-associated lethality. A preliminary evaluation of this combination was conducted in a Phase Ib study, which revealed initial signs of activity. However, the study also identified the presence of overlapping haematological toxicity, which was identified as a significant limitation [233].

Many cancers exhibit dysregulated signalling through the insulin-like growth factor (IGF) axis, leading to activation of type 1 IGF receptors (IGF-1Rs) and variant insulin receptors (INSRs). These receptors transmit signals primarily through the PI3K-AKT-mTOR and MEK-ERK pathways [234]. Via these downstream effectors, IGFs promote cell cycle progression, tumour cell proliferation, and resistance to apoptosis [234,235,236]. IGF-1R blockade sensitizes human tumour cells to ionizing radiation and cytotoxic agents [237,238,239,240,241]. Furthermore, studies have shown that the depletion or inhibition of IGF-1R can delay the repair of IR-induced DNA double-strand breaks and impair both homologous recombination and non-homologous end joining [239,241]. At the molecular level, IGF signalling is essential for DNA replication as it governs ribonucleotide reductase-mediated synthesis (RRMS) [242] and controls dNTP availability [243]. By acting via both PI3K-AKT and MEK-ERK-JUN pathways, IGF-1 upregulates RRM2 transcription [242]. When IGF activity is blocked, cells undergo tolerable RS, which exposes them to a therapeutic vulnerability [170]. Thus, IGF-1R inhibited or depleted cells downregulate RRM2 and dNTP supply, delaying replication fork progression, activating ATR/CHK1 and the replication checkpoint, and suppressing new origin firing [242], all key hallmarks of RS [244]. The tolerable RS observed in IGF-inhibited cells is exacerbated by the concomitant inhibition of CHK1, which leads to a substantial depletion of the RRM2 protein. This is consistent with the established role of CHK1 in maintaining E2F1-mediated RRM2 transcription and counteracting CDK-mediated RRM2 degradation [122].

Metabolic drug screens further suggest that metabolic targets more broadly sensitize tumour cells to CHK1 inhibition. A study of small molecule metabolism modulators revealed a combination of activities between an iCHK1 and chloroquine or the lactate dehydrogenase (LDHA/LDHB) inhibitor GSK 2837808A. Chloroquine, a lysosome and autophagy inhibitor, enhanced iCHK1-induced DNA damage and cell death by blocking metabolic adaptation pathways and increasing oxidative stress. LDHs play a central role in tumour cell metabolism by catalysing the conversion of pyruvate to lactate, thereby regenerating NAD^+^ required to sustain high glycolytic flux under aerobic conditions (i.e., Warburg effect) [245]. GSK2837808A induces metabolic reprogramming consisting of decreased cytosolic glucose and increased Krebs cycle and mitochondrial activity, which in turn leads to ROS production and subsequently DNA damage [2]. These metabolic adaptations allow synergism of GSK 2837808A with CHK1 inhibition [2].

Taken together, the observations gathered from these data indicate, on one hand, that inhibition of metabolic proteins or specific cellular pathways regulating metabolism can directly or indirectly affect genome integrity. Indeed, most—if not all—of the metabolic inhibitors or analogues mentioned above induce DNA damage either directly or indirectly (e.g., by blocking PAR metabolism or interfering with nucleotide synthesis and availability). In the event of CHK1 activation, cells can counteract these stresses. However, in the presence of an iCHK1, cells are no longer able to buffer the DNA lesions induced by such treatments. Conversely, CHK1 inhibition has been demonstrated to expose latent metabolic vulnerabilities in cancer cells, with the potential for metabolic stress to enhance iCHK1 sensitivity.

### 8.3 Limitations and Potential of CHK1 Inhibitors

The data discussed above indicate that several iCHK1 have entered early-phase clinical trials over the past two decades, where they have been evaluated either as monotherapy or, more commonly, in combination with DNA-damaging agents in Phase I/II studies. Importantly, none of these compounds has progressed successfully to Phase III clinical development, underscoring the nascent but evolving state of iCHK1 development [200,201,202].

As single agents, iCHK1 demonstrated measurable antitumour activity, particularly in molecularly defined subsets of tumours characterized by defects in the DDR, high levels of RS, or TP53 mutations. However, despite the mechanistic rationale and some clinical responses, the overall efficacy of monotherapy has been modest and frequently constrained by dose-limiting hematologic toxicities. A more promising strategy has emerged from combination regimens that exploit synthetic lethality or augment DNA damage beyond repair thresholds.

Significant challenges remain in the clinical advancement of iCHK1. Monotherapy efficacy appears restricted to specific subgroups of patients. Combination regimens represent the most extensively explored approach to date. CHK1 inhibition synergizes with DNA-damaging chemotherapeutics by abrogating cell cycle checkpoints, thereby enhancing cytotoxicity. Indeed, SRA737 combination with gemcitabine, GDC-0575 combination with gemcitabine, and prexasertib in combination with platinum-based agents like cisplatin have demonstrated improved activity compared to monotherapy. Optimal synchronization of CHK1 inhibition with DNA-damaging therapies requires further refinement, including studies evaluating sequential versus concurrent dosing strategies [215,216]. However, these combinations also exacerbate toxicity, particularly hematologic suppression, highlighting the importance of optimized scheduling and dose modulation to achieve a tolerable therapeutic index in patients [200,201,210].

Across clinical trials, the most prominent adverse effects reported are hematologic toxicities and, more rarely, cardiotoxicity [218]. In early studies, high-grade neutropenia/leukopenia and anaemia were among the most frequently observed adverse events, together with other dose-limiting toxicities that ultimately led to the discontinuation of clinical development programs [246,247]. The mechanistic basis of these CHK1-dependent hematologic toxicities is closely linked to the essential role of CHK1 in haematopoiesis. Indeed, CHK1 activity has been shown to be critical for normal blood cell development and homeostasis and to be indispensable for the survival and expansion of hematopoietic stem and progenitor cells, as pharmacological inhibition or genetic loss of CHK1 in these cells induces apoptosis and irreversible loss of haematopoiesis [100]. Given that hematopoietic progenitors are among the most rapidly proliferating normal cell populations, CHK1 inhibition in this compartment is likely to contribute directly to the clinically relevant hematologic toxicities observed in patients treated with iCHK1. In conclusion, available evidence indicates that the failure of iCHK1 to progress to Phase III clinical trials is primarily due to hematologic toxicities arising from the essential biological functions of CHK1, rather than from kinase-independent CHK1 activities.

iCHK1 are specifically designed to block the kinase activity of the protein, and only limited evidence supports physiological roles of CHK1 that are independent of its catalytic function. For instance, CHK1-dependent replication fork progression on damaged DNA through interaction with PCNA has been reported to occur independently of its kinase domain, suggesting the existence of non-catalytic functions in certain replication-associated contexts [248]. However, these kinase-independent activities appear to be highly specialized, observed in specific experimental systems, and do not represent the dominant functions of CHK1 in normal cell cycle regulation. Importantly, such non-kinase roles have not been linked to clinical toxicity or to differences in therapeutic window in humans and remain mechanistic observations at the cellular level rather than drivers of dose-limiting toxicities in patients [248].

Ongoing preclinical and clinical studies will further illuminate the role of CHK1 in cancer therapy and help refine treatment strategies, leading to improved outcomes for patients with advanced malignancies. A crucial component for the advancement of iCHK1 in clinical practice is the rigorous identification of biomarker(s), which could address the use of iCHK1 towards specific patient cohorts and enable personalized treatment regimens while minimizing adverse side effects [218].

Although an in-depth discussion of potential biomarkers for the use of iCHK1 in clinical trials is beyond the scope of the present work, here we have highlighted the role of CHK1 as a metabolic and immunological regulator, showing the mechanistic interaction among CHK1 and metabolic and immunomodulatory pathways (e.g., PGAM-glycolysis; cGAS/STING). Thus, it is tempting to speculate that proteins involved in metabolic and immune response could serve as selective biomarkers for patient stratification that could address tailored strategies for cancer co-treatments.

## 9 Future Perspectives

While CHK1 inhibition exploits the dependency of cancer cells on this kinase for RS management and survival, resistance remains a significant obstacle. One of the most critical mechanisms underlying this resistance is metabolic plasticity, which enables tumour cells to bypass CHK1-regulated pathways and adapt to therapeutic stress. This adaptive process is multifaceted. First, cancer cells can activate compensatory checkpoint pathways such as ATR, CHK2, or WEE1, maintaining cell cycle control and DNA repair despite CHK1 inhibition. Second, cancer cells frequently reprogram their metabolism toward alternative energy sources, including oxidative phosphorylation, fatty acid oxidation, and glutaminolysis, to offset the loss of glycolytic flux and sustain biosynthetic demands. Third, the activation of antioxidant systems, such as glutathione synthesis and NRF2 signalling, helps neutralize ROS accumulation typically induced by CHK1 blockade. Additionally, cancer cells can rewire glycolytic pathways by bypassing the CHK1:PGAM1 interaction, upregulating enzymes like PKM2 and LDHA, or increasing lactate export to preserve the Warburg phenotype. This metabolic flexibility is further reinforced by transcriptional reprogramming through stress-responsive factors such as HIF-1α and MYC, which restore glycolysis and anabolic pathways independently of CHK1 signalling. Finally, tumour cells may exploit their microenvironment by engaging in nutrient scavenging strategies, including autophagy and macropinocytosis, or leveraging stromal interactions to sustain growth under therapeutic pressure.

Understanding these mechanisms is essential for designing next-generation iCHK1 and combination strategies. Targeting metabolic adaptations alongside checkpoint inhibition could prevent resistance and enhance therapeutic efficacy, paving the way for more durable responses in cancer treatment. The contrasting roles of CHK1 in normal versus cancer cells create a unique opportunity for targeted therapy. Exploiting the cancer cells’ hyperdependency on CHK1 allows clinicians to define the therapeutic window for CHK1 inhibition by designing treatment regimens that maximise tumour killing while minimising harm to healthy tissues. In normal, non-proliferative cells, CHK1 activity and expression are moderate, primarily acting as a safeguard during rare division cycles. CHK1 inhibition in these cells induces reversible cell cycle arrest, with recovery mediated by ATR and p53 pathways. Although transient neutropenia may occur, HSCs tolerate partial CHK1 loss without long-term depletion, enabling intermittent dosing strategies to minimize toxicity. Conversely, cancer cells exhibit chronic RS and overexpress CHK1 (5–10× higher than normal), relying on it for replication fork stability and metabolic adaptation, including glycolysis and PPP flux for redox control. CHK1 inhibition in tumours triggers irreversible replication fork collapse, ROS overload, and apoptosis, particularly in p53-deficient or homologous recombination-defective contexts. Combination therapies, such as iCHK1 with gemcitabine, further enhance selectivity. These differences define a therapeutic window that allows effective tumour targeting while sparing normal tissues. Additionally, combining iCHK1 with other targeted agents, including immune checkpoint blockade therapies, holds promise to enhance antitumour efficacy and counteract drug resistance.

CHK1 also exerts a pivotal role in the epigenetic regulation of cell differentiation, functioning as a critical checkpoint that prevents premature exit from the cell cycle and modulates chromatin states. Maintenance of pluripotency or progenitor states is enabled by the process of histone phosphorylation and checkpoint activity, which in turn ensures proper lineage commitment. The role of CHK1 in epigenetic regulation and differentiation offers key therapeutic targets, especially in cancer stem cells and differentiation disorders. Inhibitors capitalise on synthetic lethality in tumours exhibiting high CHK1 activity or RS, while sparing normal cells.

## 10 Conclusions

Overall, data here reported indicate that: (i) inhibiting specific metabolic proteins that support the metabolic maintenance of genome stability, together with the parallel inhibition of CHK1 produces a synergistic antiproliferative effect, as these interventions simultaneously target two pathways that converge on the preservation of genome integrity; and (ii) CHK1 inhibition can itself disrupt metabolic pathways, thereby revealing specific metabolic vulnerabilities that can be further exploited through the inhibition of selected metabolic proteins. Taken together, this evidence highlights a dual opportunity for innovative therapeutic strategies based on co-treatments targeting both CHK1 and metabolic and/or immunological enzymes. Taken together, this evidence highlights a dual opportunity for innovative therapeutic strategies based on co-treatments targeting both CHK1 and metabolic enzymes.

## Data Availability

Not applicable.
